# Non-Stationarity in the “Resting Brain’s” Modular Architecture

**DOI:** 10.1371/journal.pone.0039731

**Published:** 2012-06-28

**Authors:** David T. Jones, Prashanthi Vemuri, Matthew C. Murphy, Jeffrey L. Gunter, Matthew L. Senjem, Mary M. Machulda, Scott A. Przybelski, Brian E. Gregg, Kejal Kantarci, David S. Knopman, Bradley F. Boeve, Ronald C. Petersen, Clifford R. Jack

**Affiliations:** 1 Department of Neurology, Mayo Clinic, Rochester, Minnesota, United States of America; 2 Department of Radiology, Mayo Clinic, Rochester, Minnesota, United States of America; 3 Department of Psychiatry/Psychology, Mayo Clinic, Rochester, Minnesota, United States of America; 4 Department of Biomedical Statistic and Informatics, Mayo Clinic, Rochester, Minnesota, United States of America; Beijing Normal University, China

## Abstract

Task-free functional magnetic resonance imaging (TF-fMRI) has great potential for advancing the understanding and treatment of neurologic illness. However, as with all measures of neural activity, variability is a hallmark of intrinsic connectivity networks (ICNs) identified by TF-fMRI. This variability has hampered efforts to define a robust metric of connectivity suitable as a biomarker for neurologic illness. We hypothesized that some of this variability rather than representing noise in the measurement process, is related to a fundamental feature of connectivity within ICNs, which is their non-stationary nature. To test this hypothesis, we used a large (n = 892) population-based sample of older subjects to construct a well characterized atlas of 68 functional regions, which were categorized based on independent component analysis network of origin, anatomical locations, and a functional meta-analysis. These regions were then used to construct dynamic graphical representations of brain connectivity within a sliding time window for each subject. This allowed us to demonstrate the non-stationary nature of the brain’s modular organization and assign each region to a “meta-modular” group. Using this grouping, we then compared dwell time in strong sub-network configurations of the default mode network (DMN) between 28 subjects with Alzheimer’s dementia and 56 cognitively normal elderly subjects matched 1∶2 on age, gender, and education. We found that differences in connectivity we and others have previously observed in Alzheimer’s disease can be explained by differences in dwell time in DMN sub-network configurations, rather than steady state connectivity magnitude. DMN dwell time in specific modular configurations may also underlie the TF-fMRI findings that have been described in mild cognitive impairment and cognitively normal subjects who are at risk for Alzheimer’s dementia.

## Introduction

Functional magnetic resonance imaging (fMRI) conducted without a predetermined experimental condition (sometimes referred to as resting-state fMRI), is emerging as a powerful tool for investigating the intrinsic organization of large portions of the brain into networks of synchronized activity [1]. Network information is found by analyzing low-frequency oscillations (<0.1 Hz) in the blood-oxygenation level dependent (BOLD) signal. These oscillations are readily observed with MRI systems available at most medical centers. The absence of an experimentally predetermined task in these studies has led to the popularization of the term resting-state fMRI to refer to the technique, and the term resting-state networks to refer to the identified brain networks. However, the networks identified using resting-state fMRI are also identified when applying the same analysis techniques to task-based fMRI experimental designs when the brain is not at “rest” [2]. Therefore, the technique is more accurately referred to as task-free fMRI (TF-fMRI), and the identified networks as intrinsic connectivity networks (ICNs) [3]. Removing “rest” from these terms more accurately captures the dynamic nature of the functional connectivity that characterizes these large-scale networks [4], as the brain is never truly at “rest.”

Task-free fMRI has great potential for serving as a biomarker for neurologic illness, however high variability in network measures [5] may necessitate long scanning times to distinguish an individual from the group [6]. We hypothesize that some of this variability, rather than representing noise in the measurement process [7], is related to a fundamental feature of connectivity within ICNs, which is its non-stationary nature. Some of this variability is undoubtedly related to the variability in brain states at the time of the experimentally unconstrained scanning session. Unconstrained brain activity is inherent in the TF-fMRI experimental paradigm and this leads to variability in measures of the organization of large-scale brain networks. This implies that varying brain states will affect measures of network organization, which has led some researchers to suggest restraining possible brain states by administering a task [6]. However, introducing a task reduces the appeal of the ease of TF-fMRI acquisition and does not necessarily circumvent the need for understanding the effects of the non-stationary nature of brain states on measures of network connectivity.

The non-stationary nature of ICNs during an experimentally unconstrained condition has been observed using magnetoencephalography [8]. It has also recently been shown that ICNs are related to shifting brain microstates measured via electroencephalography (EEG) [9], which are characteristically non-stationary. Yet, little is known about the variability in the organization of ICNs over time in TF-fMRI data. However, a great deal has been learned about the presumed stationary network properties of the brain via application of graph theoretical analysis of TF-fMRI data (see [10] for a review of complex network analysis methodology).

Graph theoretical analyses have demonstrated that the network architecture of the brain has scale-free small-world network organization [11], [12] and has a degenerate [13] hierarchically-organized [14] modular architecture. The segregation of regions within a graph into densely interconnected groups defines a networks modular structure, and measures of this structure are referred to as measures of modularity. The regions of the brain comprising these modules have similarities across studies, and bear resemblance to the most common networks identified as ICNs (e.g. visual, motor, task-negative and task-positive regions [15]). Given the non-stationarity in ICN, we hypothesize that the topographic organization of the brain’s modular architecture also varies over time, which may explain variability in the reported modular assignments within separate datasets [16]. If this hypothesis is validated, then the conceptualization of TF-fMRI modular architecture as non-stationary would be consistent with a recent report of the modular non-stationarity during task-based fMRI [17], thus further linking observations of the organization of low-frequency fluctuations during “rest” and task.

Understanding the dynamic network architecture in the human brain is a prerequisite to understanding how neurological disease alters this time-dependent topology. We have recently demonstrated that the age-related changes in ICNs are similar to the changes observed in Alzheimer’s disease (AD) [18], and other investigators have shown that the modular organization of the brain is also affected by aging [19]. Therefore, it is important to study the normal modular architecture of the population at risk for developing the neurologic illness of interest. The population based sampling of healthy older subjects in the Mayo Clinic Study of Aging (MCSA) [20] is ideally suited to define normal network topology in the cognitively normal population at risk for common neurodegenerative illnesses of the elderly, namely AD dementia. For these same reasons it is important to atlas the brain functionally [6] in this population as well.

This paper is organized into three major subsections. First, we constructed a population-based functional atlas. This was done by performing a large (N = 892) high dimensional independent component analysis (ICA) decomposition of TF-fMRI data from cognitively normal (CN) subjects drawn from the MCSA. This ICA decomposition was used to atlas the brain into 68 functional regions, which are categorized based on ICA network of origin, anatomical locations, and a functional meta-analysis. Second, we developed dynamic functional metrics to capture the non-stationary nature of the brain’s network topography. This was done by constructing dynamic graphical representations of brain connectivity (between the atlas defined functional regions) using a sliding time window correlation for each subject and then characterizing these graphs using recently developed graph metrics for modularity on fully connected and weighted networks [13]. Lastly, we evaluated whether non-stationarity in this modular organization was related to the reciprocal changes in connectivity between the anterior default mode network (aDMN) and posterior default mode network (pDMN) we have previously observed in subjects with AD dementia [18].

## Methods

### Subjects

Subjects enrolled in the Mayo Clinic Study of Aging (MCSA), a prospective, population based study of randomly selected residents of Olmstead County, Minnesota, who had undergone TF-fMRI and passed quality control (see below) at the time of this study were included in this study (n = 892). The MCSA cohort is composed of non-demented subjects age 70–90 plus and is balanced on gender. Only cognitively normal (CN) MCSA subjects were used in this first step in our analyses (Table 1). The details of MCSA subject recruitment and design are detailed in a previous report [20]. In summary, subjects undergo in-person evaluations by nurses, physicians and neuropsychologists, during which they gathered risk factor assessments including structured neurological exams with mental status screening and routine neuropsychological evaluation including tests measuring memory, language, executive function and visuospatial skills. A final diagnosis for each subject is made during a weekly consensus conference involving all study faculty.

**Table 1:** Cognitively normal cohort characteristics.

	Study participantsn = 892	Study non-participantsn = 945	MCSA cognitively normal participantsn = 1837
No. of females (%)	438 (49)	472 (50)	910 (50)
Age, (q1, q3)	79 (75, 83)	81 (76, 85)	80 (76, 85)
Short Test of mental status (q1, q3)†	35 (33, 37)	34 (32, 36)	34 (33, 36)

The median (IQR) are reported for the continuous variables.

† 40 subjects missing the Short Test of mental status; 13 missing from the study participants and 27 from the study non-participants.

We also evaluated subjects with AD dementia (n = 28) in our study. All met National Institute of Neurological Disorders and Stroke and the Alzheimer’s Disease and Related Disorders Association (NINDS-ADRDA) criteria and were participants of the Mayo Clinic Alzheimer’s Disease Research Center.

### Ethics Statement

All participants, or appropriate surrogates, provided written informed consent for participation. The Mayo Clinic Institutional Review Board approved the study and the consenting processes.

### Task-Free fMRI Data Acquisition and Preprocessing

Task-free fMRI data were acquired using a General Electric 3 T Signa HDx scanner, 8 channel head coil, gradient EPI, TR = 3000 ms, TE = 30 ms, 90° flip angle, 21 cm field of view, 64×64 in-plane matrix, slice thickness 3.3 mm without gap, and 103 or 113 volumes were obtained. Subjects were instructed to keep their eyes open during scanning. All TF-fMRI data sets with greater than 3 mm of translational movement, 3° of rotational movement, or that failed visual inspection for obvious artifacts were excluded from analysis (2.4% of studies did not pass these quality control measures). Preprocessing and data analysis was performed utilizing a combination of the Statistical Parametric Mapping (SPM5) software (http://www.fil.ion.ucl.ac.uk/spm/software/spm5/) (Wellcome Department of Cognitive Neurology, University College London, UK), the Resting-State fMRI Data Analysis Toolkit (REST) v1.5 (http://www.restfmri.net) [21], Data Processing Assistant for Resting-State fMRI (DPARSF) v2.0 (http://www.restfmri.net) [22], group ICA of fMRI toolbox (GIFT) software v2.0 c (http://icatb.sourceforge.net) [23], brain connectivity toolbox (http://www.brain-connectivity-toolbox.net) [10], and in-house developed software implemented in MATLAB v7.11 (Mathworks Inc., Natick, MA, USA).

Preprocessing steps included discarding the first 3 volumes to obtain steady state magnetization (sequences with 113 volumes were also truncated so all sequences had 100 remaining volumes for analysis), realignment, slice time correction, normalization to SPM5 EPI template, smoothing with 4 mm full-width half maximum Gaussian kernel, linear detrending to correct for signal drift, and 0.01–0.08 Hz bandpass filtering to reduce non-neuronal contributions to BOLD fluctuations. In addition, regression correction for spurious variables included rigid body transformation motion effects, global mean signal, white matter signal and cerebral spinal fluid signal [24], [25].

### Construction of Population Based Atlas-using the Time Series of 892 CN Subjects

#### Independent component analysis

**Figure 1:**
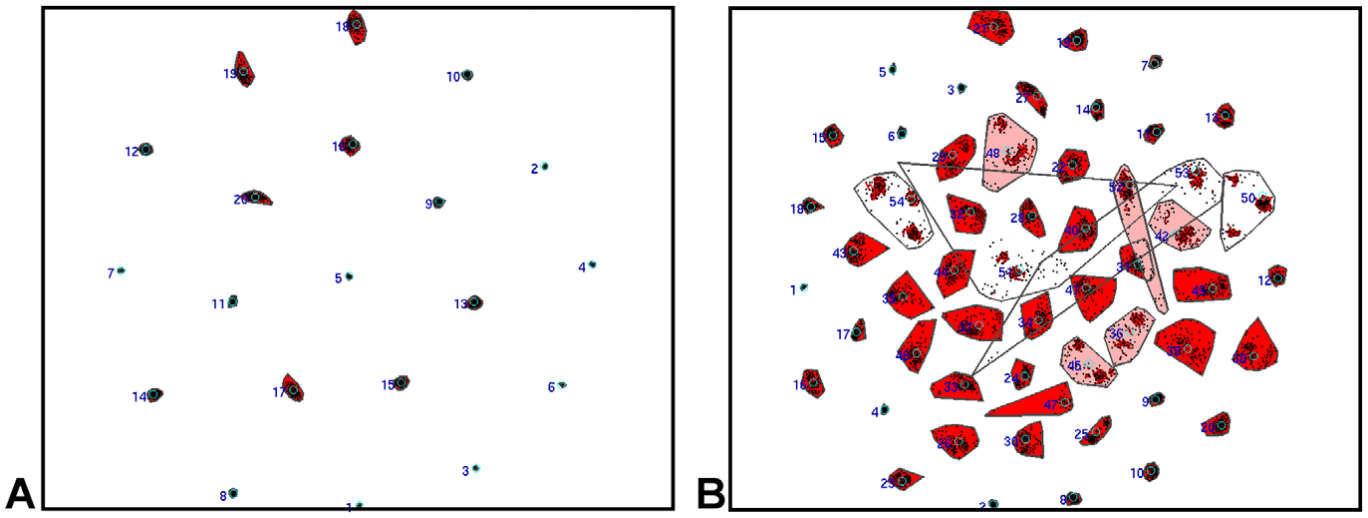
Similarity between independent component estimates as a 2D CCA projection. The similarity between the independent components identified in each of the 100 iterations of the group ICA for both the low-(A) and high-dimensional (B) runs. Points represent individual runs, grey convex hulls represent a cluster of the same independent component (labeled with component number), cyan circles represent the best estimate of the independent component (centrotype), red shading indicates average intra-cluster similarity exceeds 0.9, and pink shading indicates average intra-cluster similarity is between 0.8 and 0.9. If intra-cluster similarity is bellow 0.9, red lines are drawn between runs that have similarity greater than 0.9. Compact clusters are indicative of high run-to-run similarity in the ICA solution for that component. 2D-two dimensional, CCA-curvilinear component analysis, ICA-independent component analysis.

**Figure 2:**
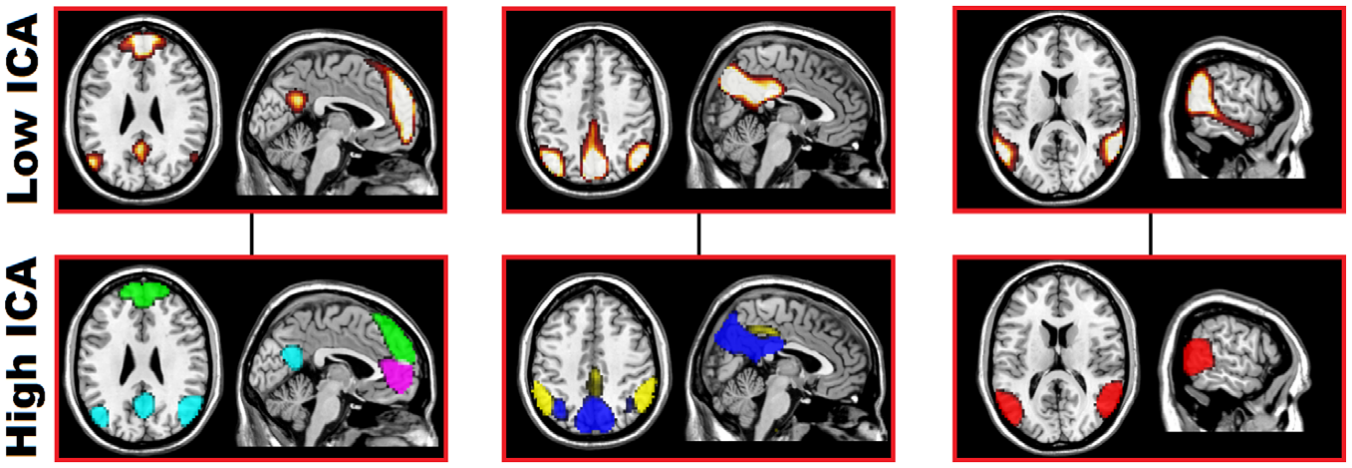
Default mode sub-networks identified with the low- and high-dimensional independent component analysis of 892 cognitively normal subjects. The DMN sub-networks identified with low- (top) and high-dimensional (bottom) ICA displayed at Z>7 overlaid on anatomical reference. The colored high-dimensional independent components were split from their low-dimensional counterpart displayed above; cyan-vDMN, green-adDMN, pink-avDMN, blue-pDMN, yellow-dDMN, red-tDMN. DMN-default mode network, ICA-independent component analysis, vDMN-ventral default mode network, adDMN-anterior dorsal default mode network, avDMN-anterior ventral default mode network, pDMN-posterior default mode network, dDMN-dorsal default mode network, tDMN-temporal default mode network.

The first step for the construction of the population based atlas was identification of ICNs in the population. We used spatial group ICA method of GIFT with both a low-dimensional estimation of 20 independent components and a high-dimensional estimation of 54 components to identify the ICNs in the population. The number of components chosen for the high-dimensional ICA was based on the components estimation procedure available in the GIFT software package. To evaluate stability and obtain the centrotype estimate of the independent components, both the high and low-dimensional group ICA analyses were run with 100 iterations using the ICASSO [26] function within the GIFT software package (Figure 1). The aggregated group component maps were scaled to Z values. Every component was visually inspected, and similar networks in both high- and low-dimensional ICA were identified to assist in naming the high-dimensional components which were subsets of the lower dimensional components (Figure 2). Artifactual components were visually identified during this processes and discarded. Each high-dimensional independent component was then assigned a name based on a combination of ICA network of origin, anatomical locations, and the functional meta-analysis result (see below). All further analyses are conducted using the high-dimensional ICA components.

#### BrainMap functional meta-analysis

**Figure 3:**
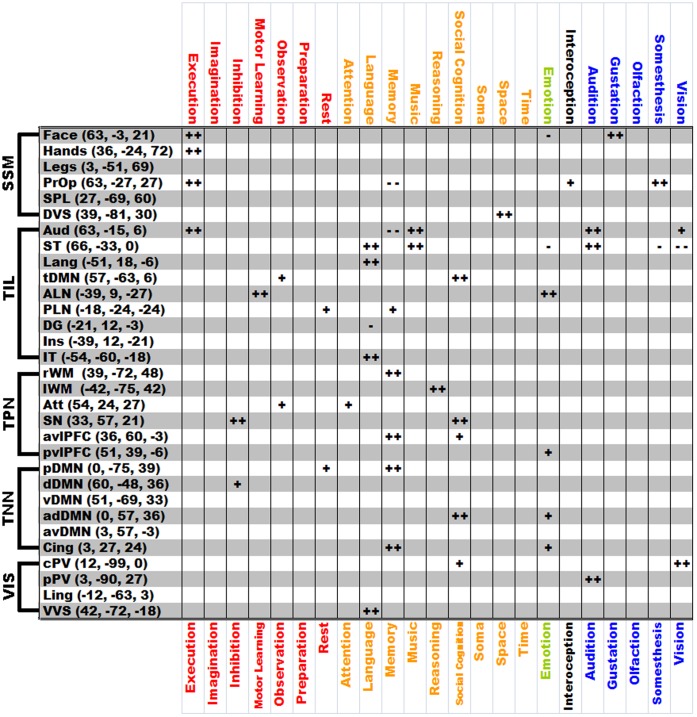
BrainMap functional meta-analysis. The results of the BrainMap functional meta-analysis are displayed for each of the 31 ICNs identified in the high-dimensional ICA in 892 cognitively normal subjects. The ICNs are grouped by final modular assignment identified on the left (the language network ICN was included in TIL for this grouping). MNI coordinates for the peak Z-score are listed in parenthesis after the ICN abbreviation (names for abbreviations can be found in Table 2). The behavioral domains are color coded by BrainMap domain of origin; red-action, orange-cognitive, green-emotion, black-interoception, blue-perception. Plus signs indicate overrepresented, and minus signs indicate underrepresented, relative to the entire brain. (+, −) FDR p<0.05 (++, −−) FDR p<0.01 ICN-intrinsic connectivity network, ICA-independent component analysis, SSM-somatic sensory-motor, TIL-temporal/insular/limbic, TPN-task-positive network, TNN-task-negative network, VIS-visual, MNI-Montreal Neurologic Institute.

Next, we used the BrainMap functional meta-analysis to aid us with the nomenclature of the identified ICNs by associating each ICN with behavioral domains. Using BrainMap’s behavioral metadata we conducted a functional meta-analysis of the behavioral domains which are overrepresented (relative to the entire brain) within a 10 mm cubic region of interest (ROI) centered at the Montreal Neurologic Institute (MNI) coordinates corresponding to the peak Z-score for each of the 31 ICNs identified by the high dimensional ICA in a similar manner as outlined by Laird *et al,* 2009 [27]. BrainMap behavioral metadata is divided into broad domains including; action, cognition, emotion, interoception, and sensation (a complete list can be accessed at http://brainmap.org/scribe). The action, cognition, and sensation domains have a significant number of highly occurring sub-domains of interest, so these categories were expanded for the subsequent analysis (a complete list of these domains is listed on the top and bottom of Figure 3).

**Table 2:** Node matrix order, module assignments, names, and abbreviations.

Module Name	Node #	Name	Abbreviation	FinalAssignment	Clustering of Assignments
SSM	1	Left Face	Face_L	1	1
	2	Right Face	Face_R	1	1
	3	Left Hand	Hand_L	1	1
	4	Right Hand	Hand_R	1	1
	5	Left Leg	Leg_L	1	1
	6	Right Leg	Leg_R	1	1
	7	Left Parietal Operculum	PrOp_L	1	1
	8	Right Parietal Operculum	PrOp_R	1	1
	9	Left Superior Parietal Lobule	SPL_L	1	1
	10	Right Superior Parietal Lobule	SPL_R	1	1
	11	Left Dorsal Visual Stream	DVS_L	1	1
	12	Right Dorsal Visual Stream	DVS_R	1	1
AIL	13	Left Auditory	Aud_L	2	2
	14	Right Auditory	Aud_R	2	2
	15	Left Superior Temporal	ST_L	2	2
	16	Right Superior Temporal	ST_R	2	2
	17	Posterior Language	Lang_P	2	2
	18	Left Temporal Default Mode Network	tDMN_L	2	2
	19	Right Temporal Default Mode Network	tDMN_R	2	2
	20	Left Anterior Limbic Network	ALN_L	2	2
	21	Right Anterior Limbic Network	ALN_R	2	2
	22	Left Posterior Limbic Network	PLN_L	2	2
	23	Right Posterior Limbic Network	PLN_R	2	2
	24	Left Deep Gray	DG_L	2	2
	25	Right Deep Gray	DG_R	2	2
	26	Left Insula	Ins_L	2	2
	27	Right Insula	Ins_R	2	2
TPN	28	Left Inferior Temporal	IT_L	3	3
	29	Anterior Language	Lang_A	3	3
	30	Anterior Right Working Memory	rWM_A	3	3
	31	Posterior Right Working Memory	rWM_P	3	3
	32	Posterior Left Working Memory	lWM_P	3	4
	33	Left Attention	Att_L	3	3
	34	Right Attention	Att_R	3	3
	35	Anterior Cingulate Region of Salience Network	SN_ACC	3	3
	36	Left Lateral Region of Salience Network	SN_L_Lat	3	3
	37	Right Lateral Region of Salience Network	SN_R_Lat	3	3
	38	Left Anterior Ventral Lateral Prefrontal Cortex	avlPFC_L	3	3
	39	Right Anterior Ventral Lateral Prefrontal Cortex	avlPFC_R	3	3
	40	Left Posterior Ventral Lateral Prefrontal Cortex	pvlPFC_L	3	3
	41	Right Posterior Ventral Lateral Prefrontal Cortex	pvlPFC_R	3	3
TNN	42	Left Lateral Posterior Default Mode Network	pDMN_L_Lat	4	3
	43	Left Medial Posterior Default Mode Network	pDMN_L_Med	4	4
	44	Right Lateral Posterior Default Mode Network	pDMN_R_Lat	4	3
	45	Right Medial Posterior Default Mode Network	pDMN_R_Med	4	4
	46	Left Lateral Dorsal Default Mode Network	dDMN_L_Lat	4	3
	47	Left Medial Dorsal Default Mode Network	dDMN_L_Med	4	4
	48	Right Lateral Dorsal Default Mode Network	dDMN_R_Lat	4	3
	49	Right Medial Dorsal Default Mode Network	dDMN_R_Med	4	4
	50	Left Lateral Ventral Default Mode Network	vDMN_L_Lat	4	4
	51	Left Medial Ventral Default Mode Network	vDMN_L_Med	4	4
	52	Right Lateral Ventral Default Mode Network	vDMN_R_Lat	4	4
	53	Right Medial Ventral Default Mode Network	vDMN_R_Med	4	4
	54	Left Anterior Dorsal Default Mode Network	adDMN_L	4	4
	55	Right Anterior Dorsal Default Mode Network	adDMN_R	4	4
	56	Left Anterior Ventral Default Mode Network	avDMN_L	4	4
	57	Right Anterior Ventral Default Mode Network	avDMN_R	4	4
	58	Left Cingulate	Cing_L	4	4
	59	Right Cingulate	Cing_R	4	4
	60	Supplementary Motor Area of Language Network	Lang_SMA	4	4
VIS	61	Left Central Primary Visual	cPV_L	5	5
	62	Right Central Primary Visual	cPV_R	5	5
	63	Left Peripheral Primary Visual	pPV_L	5	5
	64	Right Peripheral Primary Visual	pPV_R	5	5
	65	Left Lingual	Ling_L	5	5
	66	Right Lingual	Ling_R	5	5
	67	Left Ventral Visual Stream	VVS_L	5	5
	68	Right Ventral Visual Stream	VVS_R	5	5

SSM-somatic sensory-motor, TIL-temporal/insular/limbic, TPN-task-positive network, TNN-task-negative network, VIS-visual.

For each ICN ROI, a chi squared test was performed to evaluate the proportion of each behavioral domains occurrence within the ROI relative to the entire brain. In order to account for the inherent multiple comparisons problem, a false discovery rate (FDR) correction was utilized, and results are reported significant at FDR corrected p<0.05 and p<0.01.

### Development of Dynamic Functional Metrics-using the Time Series of 892 CN Subjects

#### Dynamic graph construction using sliding time windows

All graph metrics used in this study were obtained from the brain connectivity toolbox (http://www.brain-connectivity-toolbox.net) [10]. In order to construct graphical representations which retain interpretability in terms of the above defined ICNs, the 31 ICNs identified by the high-dimensional ICA were used to develop 68 ROIs to be used as graph nodes. The 68 nodes were created by applying a stringent threshold to the group independent components defined by a Z-score greater than 7, which resulted in distinct clusters within each of the components. These clusters were then manually extracted using MRIcron (http://www.mccauslandcenter.sc.edu/mricro/mricron/). Clusters that crossed the midline were divided into right and left ROIs. This resulted in the creation of 68 ROI images, which are available for download at http://mayoresearch.mayo.edu/mayo/research/jack_lab/supplement.cfm. The BOLD signal within these 68 ROIs was then extracted from the pre-processed TF-fMRI data across the 100 volumes for each subject. Pearson’s correlation coefficient was then used to create the connectivity matrices. With the recent development of graph metrics applicable to fully connected and weighted graphs [13], it was not necessary to restrict our graphical representations using only positive weights and arbitrary r-value cutoffs.

**Figure 4:**
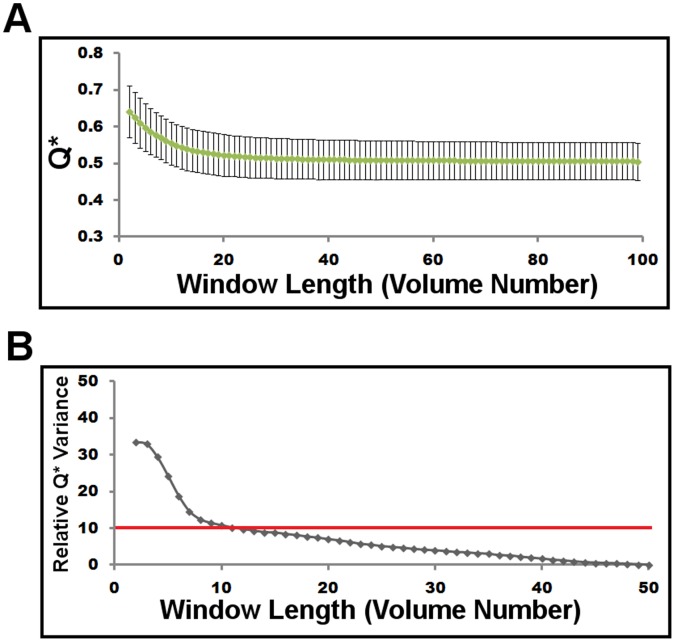
Q* versus window length in 892 cognitively normal subjects. The mean (green line) and standard deviation (error bars) of Q* are plotted for all graphs at each window length (A). The standard deviation of Q* is plotted as the percentage greater than the standard deviation present at a window length using half of the available volumes (50 volumes) and reported as the relative Q* variance (B). In order to define the shortest possible reliable odd window length, a predefined cutoff of a relative Q* variance of 10% was selected and is identified with a red line in B demonstrating the chosen window length of 11 volumes (33 seconds).

With the aim of investigating the dynamic nature of the brain’s modular structure, graphical representations were constructed within the smallest odd numbered sliding time window from which reliable modular graph metrics could be obtained (an odd number time window allows for centering of the obtained metric on one time point). To define the smallest reliable window size, we used the variability within the recently developed measure of goodness of modular partition metric, Q* [13]. Graphs with high Q* have greater than chance-expected total within module weight, and ranges from 0 to 1, and we consider high Q* to be values above the Q* of the null model of a randomized graph preserving the original graph’s degree-, weight- and strength-distribution (see [13] for a complete discussion of Q* and the null model). The variance in Q* began to stabilize around a window size of 27 seconds (9 volumes) and dropped to 10% greater than the variance present using half of the total volumes at a window length of 33 seconds (11 volumes) (Figure 4). Therefore, all subsequent analyses were conducted using a window length of 33 seconds. At this window length, 90 graphical representations of connectivity in each individual brain could be created. This resulted in 80,280 graphs for 892 subjects which were used in the subsequent modular analysis.

#### Modular analysis

For each of the 80,280 graphs, the modular assignments and Q* was calculated. A degree-, weight- and strength-distribution preserving null model was constructed for each of the 80,280 graphs and the Q* was calculated for these as well [13]. Comparisons between Q* and the null Q* distribution was conducted using the Wilcoxon two-sided rank sum test.

The number of modules each graph contained ranged from 2–5 modules at the predefined smallest reliable odd numbered window length of 33 seconds. The proportion of each was calculated across all graphs and the percent time, or modular dwell time, an individual subject spent in the modular configurations was also estimated (except for the 5 modular configuration given its infrequent occurrence across the 80,280 graphs). In order to examine the effect of window length on module number, the modular proportion was also calculated across all possible window lengths.

Next, we assigned each of the 68 ROIs to a single module, allowing for subsequent graphical analysis. Given that the modular assignment number is arbitrary and not necessarily related from graph to graph (numbers denoting groups ranging from 1–5) and the non-stationarity in modular composition, we grouped together nodes that tended to get the same modular assignment number across the different modular configurations in order to define a “meta-modular” structure. For example, visual system nodes may receive a modular assignment of module label 1 within a 2 modular configuration, while other nodes are then assigned module label 2. While in another 2 module configuration, the visual and auditory nodes are assigned module label 2 while other nodes are assigned module label 1. However, across all possible modular configurations, the visual nodes were typically always assigned to the same module, while other nodes’ co-assignment with the visual nodes was more variable. Given this variability, we captured the “meta-modular” configuration using Ward’s method of agglomerative hierarchical clustering applied to the modular assignments for all 80,280 graphs. Since the highest observed modular configuration was 5, we report the meta-modular assignment at a maximum cluster cutoff of 5. However, for the final modular assignment we also considered the ICN of origin. For visualization, the nodes were ordered within the connectivity matrices based on this final modular group assignment.

### Application to Alzheimer’s Disease

We applied the developed sliding time window graph construction technique, using the 68 ROIs as nodes with final modular assignments listed in Table 2, to a group of 28 Alzheimer’s disease patients to investigate if the non-stationary nature of the brain’s network architecture is related to differences observed in functional connectivity between AD and CN subjects, see [28] for a recent review. Specifically, we examined whether dwell time in a particular modular configuration is related to our previously reported differential effect of Alzheimer’s disease on the aDMN and pDMN [18]. To this end, we calculated the composite within-module degree Z-score for each of the DMN sub-networks in the task-negative network module (i.e. posterior, anterior, dorsal, and ventral DMN) using the brain connectivity toolbox [10].The composite score was the average score for each of the four nodes in the DMN sub-network. DMN sub-networks were considered a strong contributor to the brain state if their composite within-module degree Z-score was greater than the average (i.e. positive z-values). The percentage of time in which the sub-network strongly contributed to an individual subject’s brain state is reported as the DMN dwell time. The 28 AD subjects were matched 1∶2 on age, gender, and education to a subset of 56 CN subjects for comparison. The pair-wise comparisons of DMN dwell time for AD and CN subjects were conducted using the Wilcoxon two-sided rank sum test.

## Results

### Subjects

Given that one of the motivations for this study was to develop a baseline for investigating Alzheimer’s disease and other neurodegenerative illnesses in the elderly population, a summary of the sample characteristics of the subset of imaged CN patients used in this study relative to the entire MCSA population are reported in Table 1. The MCSA subjects with TF-fMRI were representative to the subjects that were not included for analyses. The age-, gender-, and education-matched subset of controls and AD subjects’ demographics are reported in Table 3.

**Table 3:** Comparison of DMN sub-network dwell time between Alzheimer’s and age-, gender-, and education-matched cognitive normal subjects.

	CN (n = 56)	AD (n = 28)	p-value
**No. of females (%)**	20 (35.7)	10 (35.7)	1
**Age (q1, q3)**	78 (74, 84.5)	78 (74, 84.5)	0.92
**Education (q1, q3)**	15.5 (12,18)	15.5 (12,18)	0.97
**STMS (q1, q3)**	35 (33, 37)	23.5 (21.5, 29)	<0.001
**CDR-SOB (q1, q3)**	0.0 (0.0, 0.0)	4.5 (3.0, 6.8)	<0.001
**pDMN DT (q1,q3)**	52.8 (38.3, 63.9)	42.8 (21.7, 57.8)	0.048
**aDMN DT (q1,q3)**	57.8 (33.3, 75.6)	73.9 (58.9, 87.8)	<0.001
**vDMN DT (q1,q3)**	83.9 (68.9, 91.1)	80.0 (66.7, 87.8)	0.48
**dDMN DT (q1,q3)**	46.1 (29.4, 65.6)	39.4 (23.9, 56.7)	0.39

The median, IQR, and p-values from the Wilcoxon two-sided rank sum test are reported for the age, education, STMS, CDR-SOB, and dwell time in strong DMN sub-network modular configurations for AD subjects and age-, gender-, and education-matched CN subjects (the gender proportions and the p-value of the chi-squared test between groups is reported as well). DMN dwell time is defined as the proportion of time (reported as a percentage) that a subject spends in a particular modular configuration (strong DMN sub-network configurations in this case) during the TF-fMRI scanning session.

AD-Alzheimer’s disease, STMS-short test of mental status, CDR-SOB-clinical dementia rating scale sum of boxes, aDMN-anterior default mode network, DT-dwell time, pDMN-posterior default mode network, vDMN-ventral default mode network, dDMN-dorsal default mode network, CN-cognitively normal, IQR-interquartile range, TF-fMRI-task-free functional magnetic resonance imaging.

### Population Based Atlas

#### Independent component analysis

The low-dimensional ICA revealed 15 ICNs and the high-dimensional ICA revealed 31 ICNs. The low-dimensional ICA components tended to include several of their higher-dimensional counterparts within the same independent component. This information was retained when naming the high-dimensional components. For example, the three low-dimensional DMN components and the corresponding six high-dimensional components which retain the DMN moniker are shown in Figure 2. This splitting was characteristic of other networks. The dorsal visual stream, ventral visual stream, and primary visual network for peripheral vision were all in the same low-dimensional independent component, while each had distinct independent components in the high-dimensional ICA. While the high-dimensional decomposition was finer grained, it was also more susceptible to the stochastic nature of the group ICA algorithm. This manifested in less compact clustering of the 100 repeated group ICA runs using the ICASSO procedure in the high-dimensional ICA compared to the low-dimensional (Figure 1). The MNI coordinates for the peak Z-score of each high-dimensional ICN are included in Figure 3. The aggregate component maps for both the high- and low- dimensional ICA are available for download at http://mayoresearch.mayo.edu/mayo/research/jack_lab/supplement.cfm.

#### BrainMap functional meta-analysis and network nomenclature

The BrainMap functional meta-analysis revealed a statistically significant result for 26 of the 31 ICNs (Figure 3). This information was then used to better inform the final network nomenclature. For example, the dorsal and ventral visual stream networks, which are sometimes referred to as lateral visual ICN, had a functional meta-analysis result consistent with properties associated with the dorsal and ventral processing pathways (i.e. the ventral stream was associated with the language domain and the dorsal was associated with the spatial domain, fitting with the “what” and “where” pathway distinction). The functional meta-analysis also highlighted the distinct differences in two ICNs which involved the primary visual cortex. One of these networks was strongly associated with the vision domain in the meta-analysis, while the other was below statistical significance in the visual domain but was highly significant in its association with the auditory domain. This suggests that the visual network strongly associated with audition is likely representative of the portion of the primary visual cortex dedicate to the peripheral visual field, which is commonly observed to co-activate in auditory fMRI paradigms [29]. The anatomical location of these two visual ICNs is also consistent with one accounting for the central visual field and the other with the peripheral. The final visual-related ICN did not have a significant result in the meta-analysis, however it largely followed the anatomical extent of the lingual gyrus and therefore we retained that anatomic moniker in this analysis. This naming procedure was also informed by the ICA network of origin as outlined above.

### Modular Analysis

**Figure 5:**
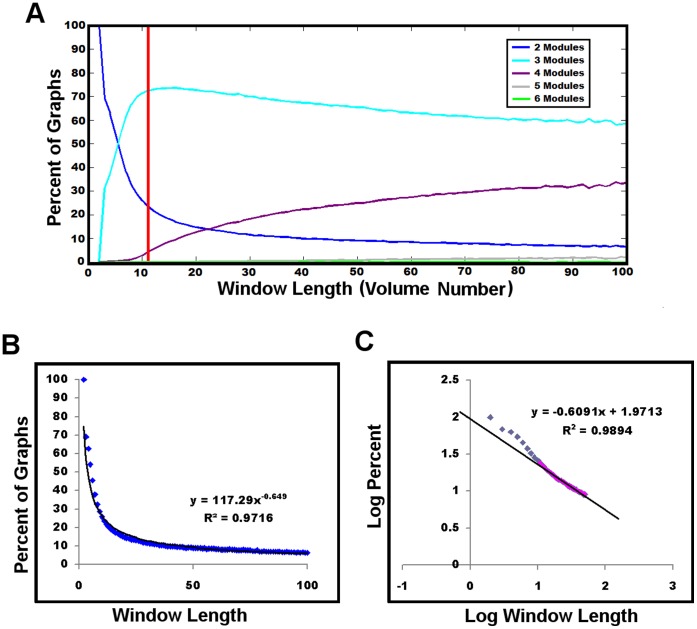
Proportion of modular configurations versus window length in 892 cognitively normal subjects. The effect of window length on the proportion of modular configurations is demonstrated by plotting the percentage of graphs with a modular configuration with a particular number of modules (2–6 modules) at each window length (A). The proportion of 2–5 modules present at the shortest reliable odd number window length of 11 (see Figure 4) is indicated by the red line. The proportion of 2 modular configurations decayed as a power-law function of window length (B). The reliable window lengths (11–50 used in Figure 4B) were linearly fit in the base 10 log-log plot of the proportion of 2 modular configurations versus window length (C). This demonstrates the estimated proportion of 2 modular configurations present at a window length of zero (instantaneous modular configuration) approaches 100 percent (y-intercept), without directly measuring the smaller unreliable window lengths (gray squares).

The effect of window length on modular proportion is significant, as would be expected in a non-stationary process (Figure 5A). Longer window lengths measure predominately 3 and 4 modular configurations, consistent with a recent report on several data sets analyzed at comparable window sizes [13]. The proportion of 2 modular network configurations decreased as a power-law function of window length (Figure 5B). Given that we could not confidently decrease the window length beyond ∼33 seconds (11 volumes), we attempted to estimate the instantaneous (i.e. window length of zero) proportion of 2 module configurations. To this end, we created a log-log plot of the proportion of 2 modular configurations versus the window lengths used in the assessment of reliability (Figure 4B). We then linearly fit the reliable window lengths (11–50 volumes) in the log-log plot and calculated the y-intercept (Figure 5C). This revealed that the estimated instantaneous proportion of 2 module configurations approached 100% (actual estimate was 93.6%). In addition, window lengths smaller than 11 in the log-log plot fall off the linear trend of the more reliable window lengths further supporting our use of the smallest possible reliable window length of 33 seconds (11 volumes).

Each of the 80,280 graphs, form the shortest reliable odd number window length of 33 seconds, displayed a highly modular architecture relative to the null model (median and interquartile range for Q* = 0.55, 0.52–0.59, and for null Q* = 0.16, 0.15–0.17, p = 0). This result indicates that the high Q* values, characteristic of dynamic brain connectivity matrices, are measuring true modularity that is consistently greater than any potential chance modular structure present in each of the 80,280 graphs.

**Figure 6:**
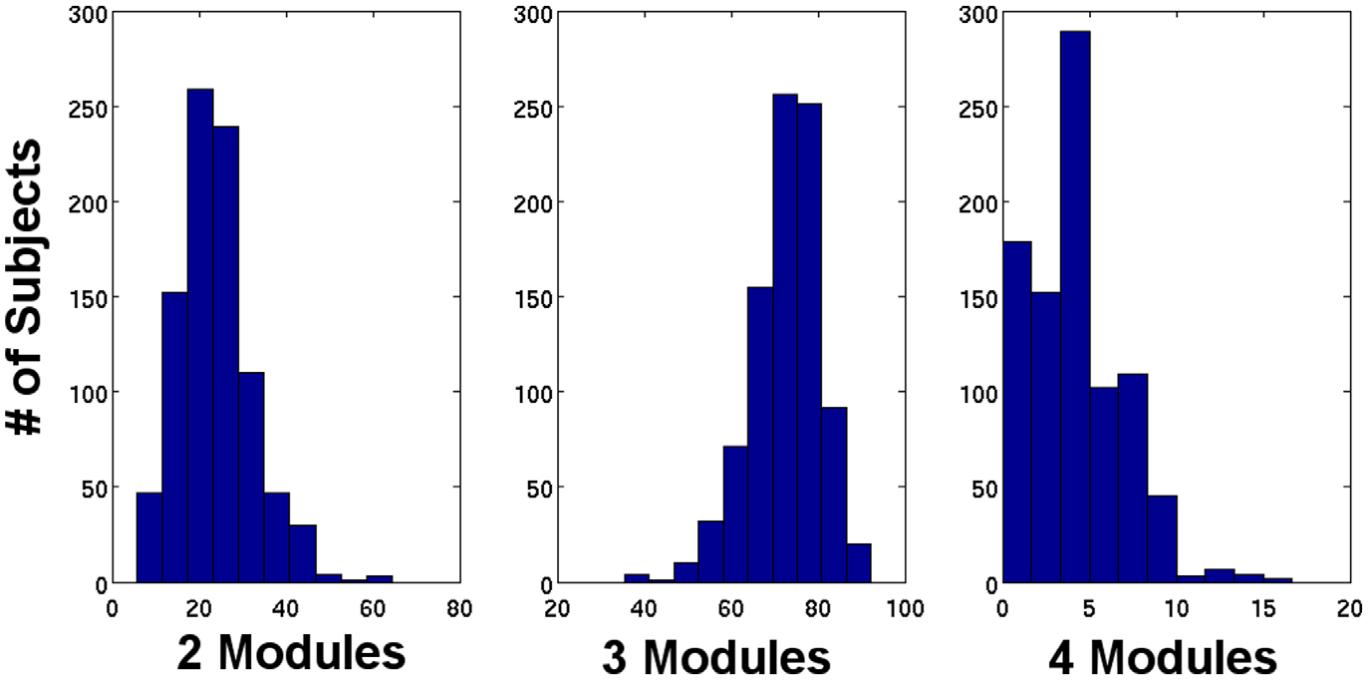
Histograms of modular dwell time in 2, 3, and 4 modular configurations at the smallest odd number reliable window length for the 892 cognitively normal subjects. The modular dwell time is defined as the proportion of time (reported as a percentage on the x-axis) that a subject spends in a modular configuration with a particular number of modules (i.e. 2,3, or 4) during the TF-fMRI scanning session.

The number of modules within each of the 80,280 graphs varied between 2 and 5, at the predefined smallest reliable odd numbered window length of 33 seconds (11 volumes), with 23.63% in a 2 module configuration, 72.38% in a 3 module configuration, 3.98% in a 4 module configuration, and less than 0.01% in a 5 module configuration (red line in Figure 5A). This distribution across all graphs was similar for the modular dwell time within the 90 graphs from a single subject’s scanning session, with each of the 892 subjects spending the majority of time during the scanning session in a brain state with either a 2 or 3 module configuration at a window length of 33 seconds (Figure 6).

**Figure 7:**
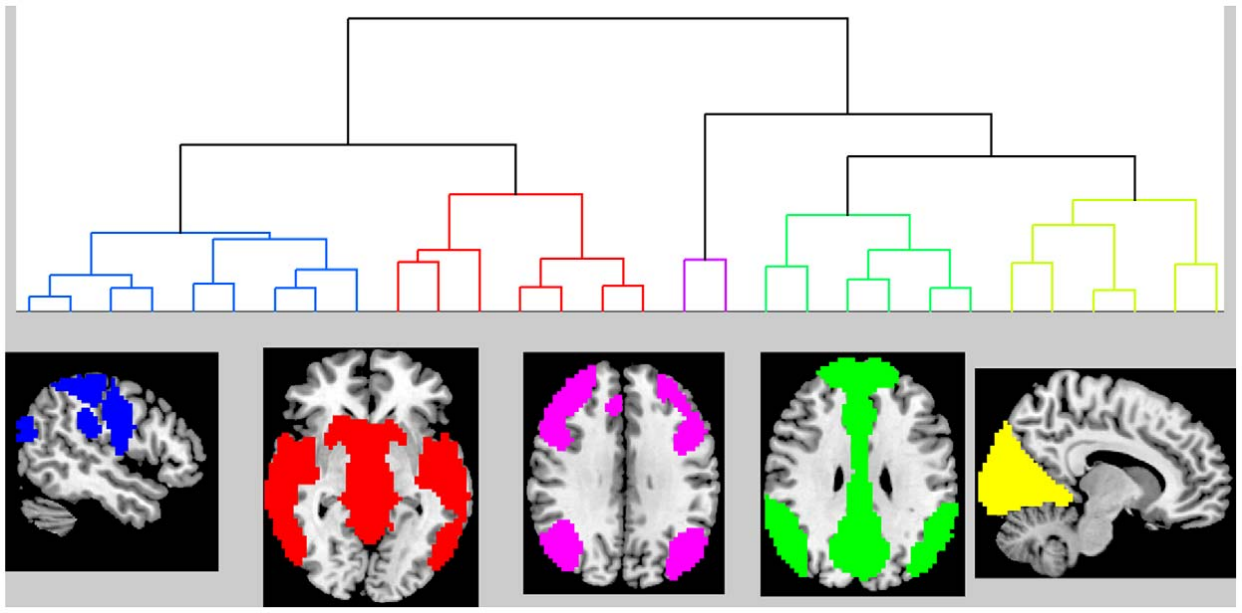
Clustering of modular assignment for the 68 regions of interest, from the 892 cognitively normal subjects. The dendrogram for the clustering of the 68 ROIs is displayed above. The colored regions in the dendrogram correspond to the similarly colored overlaid ROIs below (see Table 2 for each ROIs cluster assignment). ROI-region of interest.

**Figure 8:**
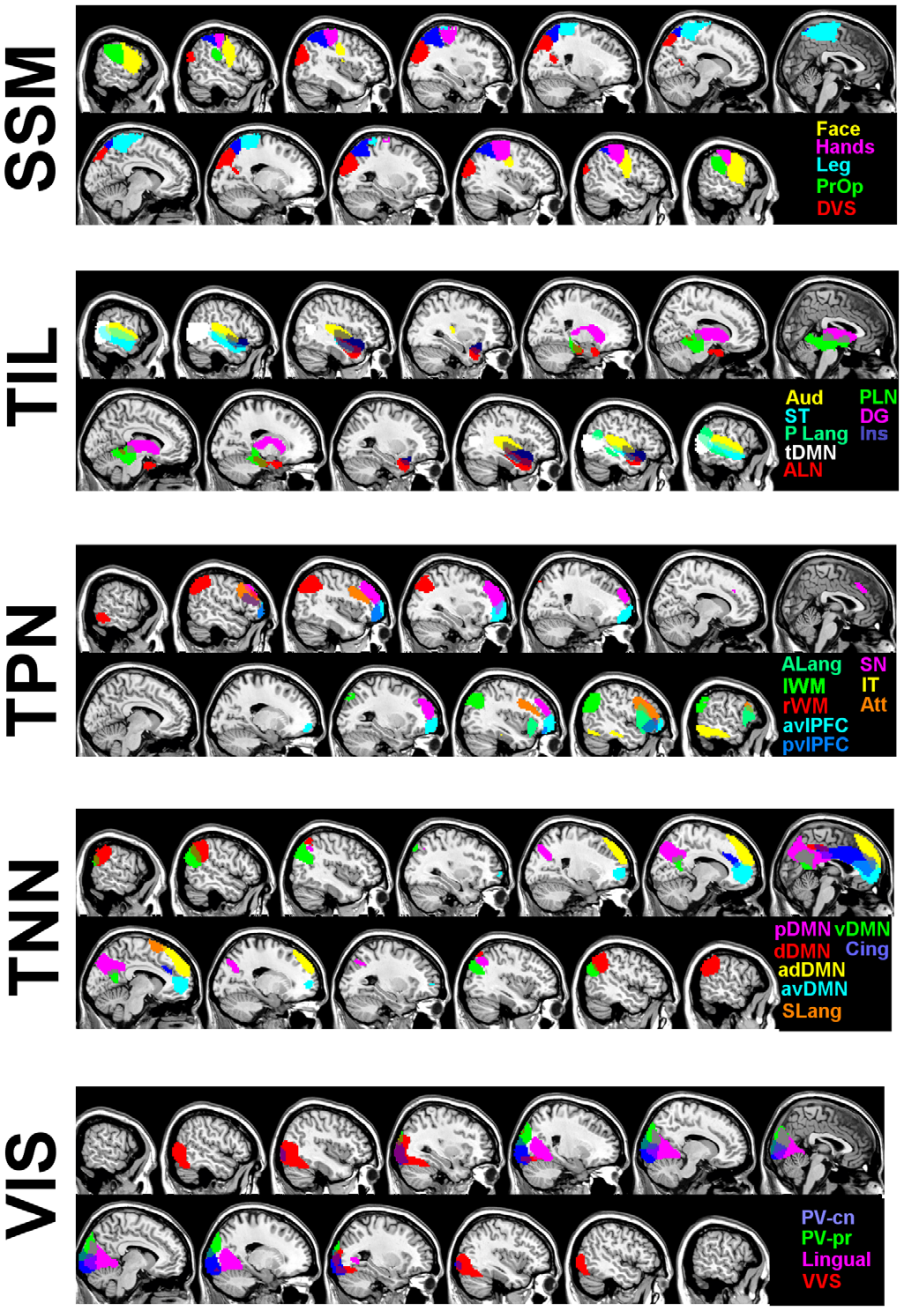
Spatial extents of the 68 ROIs divided by final module assignment, from the 892 cognitively normal subjects. The spatial extents of the 68 ROIs are overlaid on a template brain using MRIcron. The network abbreviations are color-coded in the bottom right of each panel (see Table 2 for a list of corresponding names). The module abbreviations are listed to the left of each panel. ROI-region of interest, SSM-somatic sensory-motor, TIL-temporal/insular/limbic, TPN-task-positive network, TNN-task-negative network, VIS-visual.

The agglomerative hierarchical clustering of the modular assignments into “meta-modular” groupings revealed clusters largely composed of nodes from related ICNs (Figure 7). Even though this was expected, it was further validation that nodes within an ICN behaved synchronously in graphical form as well. The “meta-modules” consisted of a somatic-sensory motor module (SSM), temporal/insular/limbic module (TIL), task-negative network module (TNN), task-positive network module (TPN), and a visual module (VIS). While it is true that most nodes clustered back with the ICN from which they were derived, there were some exceptions. The lateral parietal portions of the posterior DMN (pDMN) and dorsal DMN (dDMN) clustered with the task-positive network. We elected, however, to assign nodes derived from the same ICN to the same module for the final modular assignment. An exception was made for the language ICN because the three nodes in the language ICN were all assigned to different clusters; the anterior language node (left frontal operculum) to the TPN, the posterior language node (left temporo-parietal) to the TIL, and the supplementary motor area node to the TNN. The “meta-modular” clustering and final nodal assignments are listed in Table 2, and the spatial extents of the final 5 modular assignments are displayed in Figure 8.

**Figure 9:**
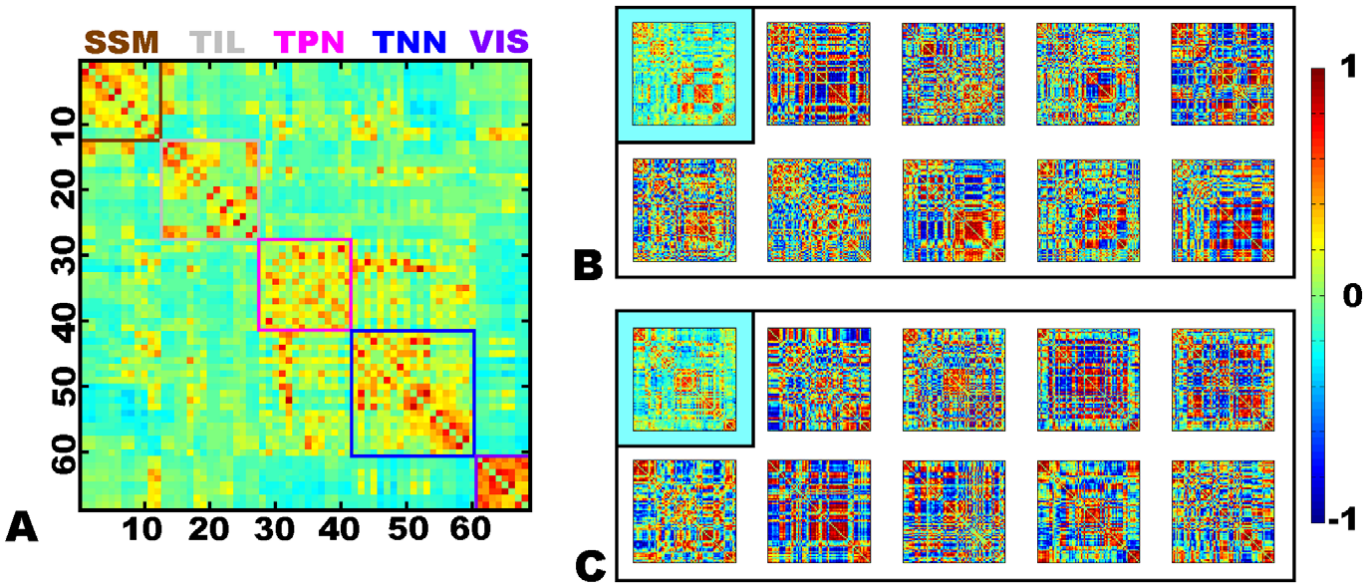
Fully connected and weighted graphs in 892 cognitively normal subjects. The fully connected and weighted graphical representation of the connectivity between the 68 ROIs at the smallest reliable odd number window length is displayed as connectivity matrices for the average of all graphs (A) and two subjects (B and C). The color bar encodes Pearson correlation strength for all 3 figure panels. The 68 ROIs are arranged by final modular assignment (see Table 2 for ROI order). The within-module connections are highlighted with color-coded boxes for each module in the average connectivity matrix (A). B and C display the average matrix for individual subjects (upper left corner inset of each panel) and every tenth frame of the sliding time window analysis follows, which increase in time from left-to-right along the top row followed by the bottom row. The videos of the entire sliding time window analysis for these two subjects are included in the supplementary material. SSM-somatic sensory-motor, TIL-temporal/insular/limbic, TPN-task-positive network, TNN-task-negative network, VIS-visual.

The average connectivity matrix for all 80,280 graphs revealed that the VIS module had strong average within module connectivity (Figure 9A). However, the dorsal visual stream also had greater average connectivity with the VIS module rather than the SSM module in which it was grouped in the cluster analysis of modular assignment. Similar discrepancies between modular clustering and the average connectivity matrix are evident in other networks as well; however there remains strong agreement between the average connectivity matrix and the final modular assignment. Still, it is clear that both the average connectivity matrix and final modular assignments do not capture the non-stationary nature of the brain’s modular architecture (Figure 9 B and C). The connectivity between nodes varied continuously across the scanning session (see Videos S1 and S2), which in turn leads to varying graph topography which does not resemble the group average. This was also true for the average connectivity matrix for an individual subject, in which the average topography was driven by the dwell time in particular modular configurations. In other words, the longer a subject spent in a particular brain state, the more it contributed to the topography of the average connectivity matrix.

### Application to Alzheimer’s Disease

To examine whether dwell time in a particular configuration is related to our previously reported differential effect of Alzheimer’s disease on the aDMN and pDMN [18], we calculated the composite within-module degree Z-score for each of the DMN sub-networks in the TNN module (i.e. posterior, anterior, dorsal, and ventral DMN). We found that Alzheimer’s subjects had lower DMN dwell time, relative to age-, gender-, and education matched controls, in brain states with strong pDMN contributions and a higher dwell time in brain states with strong aDMN contributions (Table 3) thus supporting our hypothesis that the non-stationary nature of ICNs significantly influences the average-over-time-series group differences that were observed in our previous results [18]. There was no group-wise difference in the dwell time for the dDMN and the vDMN. Similar results were also observed when comparing AD to the entire population-based cohort (results not shown).

## Discussion

Since Berger’s initial investigations into the spontaneous oscillatory patterns in the human brain [30], it has been recognized that there is an underlying pattern of organization to these oscillations that is present at all times, including the brain state of “rest”. Graph theoretical analyses of TF-fMRI data have demonstrated that this organization has scale-free small-world network organization [11], [12] and has a degenerate [13] hierarchically organized [14] modular architecture. Similar properties are present in large-scale brain networks observed using techniques with different temporal resolution [31]–[33]. In this study, we demonstrate that non-stationarity in the brain’s network topography also exists at the temporal resolution of TF-fMRI studies, and estimate that the instantaneous large-scale organization of the brain is a binary modular state. This notion has face validity because it implies that at any instant, the brain organizes itself into an “active” module that is focused on a specific functional quality, with portions of the reminder of the brain in a “non-active” state. We explored the composition of the varying topography within the context of a well characterized functional parcellation of the brain from a large population based sample of subject’s at risk for AD dementia. This allowed us to demonstrate that the non-stationary nature of the brains modular organization is related to the differences in aDMN and pDMN connectivity in AD dementia. However, even the non-stationary metric introduced here remains burdened with high variability.

Variability is a hallmark feature of measures of neural activity, prompting the development of techniques which average across multiple trials and subjects, or prolonging signal acquisition in order to reduce this variability. These techniques as applied to TF-fMRI have yet to yield a metric that is robust enough to be used as a biomarker at the individual subject level [34]. Therefore, a better understanding of the origins of the variability present in ICNs is needed. Demographics such as age and gender are sources of variability [35] as is the unconstrained nature of the task-free experimental paradigm. However, only a small reduction in variability related to the task-free experimental design is achieved with the addition of a simple task [6], and highly structured tasks typical of fMRI activation experiments still have a significant amount variability necessitating averaging across trials and subjects [36]. In addition, genetic factors such as *APOE ε*4 carrier status are also sources of variability independent of gray matter density [37] and Alzheimer’s pathology [38]. However, controlling for all of these factors will not circumvent the need for understanding the effects of the non-stationary nature of brain states on measures of network connectivity. It may indeed be the case that the variability related to the non-stationary properties of ICNs are the salient features which may distinguish AD-related alterations of connectivity.

The inherent variability in large-scale neural networks was more apparent in our high-dimensional ICA, as this analysis was more susceptible to the stochastic nature of the ICA process (Figure 1). The higher dimensional ICA was a finer-grained parcellation of the brain across all of the subjects’ average network configurations. This finer-grained solution may be the reason for the greater variability, given that we observed that the brain is organized into binary modular networks at any instantaneous point in time, with finer-grained higher-order modular configurations being observed by averaging binary states over time. The regions of the brain within any given modular organization were highly variable, but regions typically reported as ICNs seemed to form common groupings within this varying modular structure more often than not (Figure 8). This suggests that the typically observed ICN (with accompanied anticorrelations) represent an average representation of the most common binary brain configurations over the observed time period. The fact that at any given time the brain’s network topography consists of a binary modular structure, may relate to the difficulties human beings encounter while multitasking [39]. However, it should be noted that the relationship between the large-scale organization of the brain’s connectivity and cognitive performance remains uncertain. Although, our results (Figure 3) and others [2], [27], [40] indicate that ICNs observed under the task-free condition relate to observed results in highly structured task-based fMRI studies. Developing a conceptual link between TF-fMRI studies, task-based fMRI studies, and cognitive performance will improve communication of results and allow for a better understanding of the effect of neurologic disorders, such as AD, on cognition and neural networks. To this end, non-stationarity in modular composition should be considered an intrinsic property of the brain’s organization in a “task-free” state as well as “task-related” states [17].

The observed divergent changes between the aDMN and pDMN, which we previously reported using ICA and seed-based connectivity studies [18], are also present in the dwell time in strong aDMN and pDMN brain states (Table 3). Compared to CN, AD subjects had greater dwell time in strong aDMN sub-network modular configurations and less dwell time in strong pDMN configurations. Thus varying DMN dwell time in specific modular configurations, rather than steady state connectivity magnitude, seems to underlie the functional connectivity findings that have been routinely described in AD dementia. Dwell time in specific modular configurations may also underlie the TF-fMRI findings that have been described in mild cognitive impairment [41]–[44] and cognitively normal subjects who are at risk for AD dementia [37], [38], [45], [46]. It remains to be seen whether AD associated changes in non-stationary connectivity metrics are related to AD subjects transitioning into abnormal brain states, the manner in which they transition between normal brain states, or a combination of both. Future investigations into the reciprocal pattern observed in pDMN and aDMN dwell time may also help to clarify the mechanisms behind reciprocal network changes commonly observed in TF-fMRI studies [47].

While this study has observed some of the properties of the non-stationary nature of ICNs, it is yet to be shown how many configurations are possible and what the composition of those configurations might be. In this regard, future studies utilizing instantaneous frequency estimates in graph construction may be informative. This will be an important step to be taken in order to better understand how neurodegenerative illnesses affect the varying organization of the brain. In this regard, our study is partially limited by the inherent heuristics present in our analysis methodology; however the large sample size and null model gives confidence that the properties reported here are robust. We do not believe that the non-stationary nature of the brain’s complex network architecture measured with TF-fMRI can be explained simply by noise. Several features of the non-stationarity observed in this study, beyond the difference identified in AD, support a physiologically meaningful etiology.

Not only does modular dwell time vary within subjects across the scanning session, but the nodal assignment to these modules was highly variable. However, as can be readily appreciated from Video S1 and S2, the variation was regular with multiple nodes reorganizing the entire network by losing edges with one module and gaining edges with another community simultaneously. We attempted to capture some features of this dynamical process with the agglomerative hierarchical clustering analysis (Figure 7). While noise may seem like a plausible explanation for apparent non-stationarity in a node-to-node correlation, the coordinated non-stationarity present in the entire set of nodes, and the graphical metrics characterizing the global network they comprise, can not easily be attributable to noise alone. A more natural interpretation would be that the scale-free nature of the brain’s network architecture also extends to the property of non-stationarity commonly observed at higher frequencies with more direct electrophysiologic measures [8]. We hypothesize that the meta-stable brain states observed in this analysis are the low-frequency analogs of the higher-frequency microstates [9], albeit with different temporal and spatial characteristics. More work is needed to characterize these brain state configurations in their most rudimentary binary form and how they associate over time to form the higher-order network topography typically observed by averaging over long window lengths. We intend for the high- and low-dimensional decomposition of the MCSA TF-fMRI cohort and the regions of interest used for non-stationary graph construction to serve as a reference for ongoing investigations into these properties (available for download at http://mayoresearch.mayo.edu/mayo/research/jack_lab/supplement.cfm).

## Supporting Information

Video S1 **Entire sliding time window connectivity matrix for the subject in** **Figure 9B** **in the main text.** This supplementary video contains the complete sliding time window analyses for the subject displayed in Figure 7B. The color bar encodes Pearson correlation strength. The 68 ROIs are arranged by final modular assignment (see Table 2 for ROI order). The video plays at a rate of 3 frames per second with each frame representing 3 seconds, which correspond to a playback rate 9 times faster than real time.
(ZIP)

Video S2 **Entire sliding time window connectivity matrix for the subject in** **Figure 9C** **in the main text.** This supplementary video contains the complete sliding time window analyses for the subject displayed in Figure 9C. The color bar encodes Pearson correlation strength. The 68 ROIs are arranged by final modular assignment (see Table 2 for ROI order). The video plays at a rate of 3 frames per second with each frame representing 3 seconds, which correspond to a playback rate 9 times faster than real time.
(ZIP)
